# Immune checkpoint inhibitors as potential therapy for reverting T-cell exhaustion and reverting HIV latency in people living with HIV

**DOI:** 10.3389/fimmu.2023.1270881

**Published:** 2023-12-07

**Authors:** José M. Benito, Clara Restrepo, Jesús García-Foncillas, Norma Rallón

**Affiliations:** ^1^ HIV and Viral Hepatitis Research Laboratory, Instituto de Investigación Sanitaria Fundación Jiménez Díaz, Universidad Autónoma de Madrid (IIS-FJD, UAM), Madrid, Spain; ^2^ Hospital Universitario Rey Juan Carlos, Móstoles, Spain; ^3^ Department of Oncology and Cancer Institute, Fundacion Jimenez Diaz University Hospital, Autonomous University, Madrid, Spain

**Keywords:** immune checkpoint inhibitors, inflammation, cancer, HIV, immune exhaustion, HIV latency, immunotherapy, HIV cure

## Abstract

The immune system of people living with HIV (PLWH) is persistently exposed to antigens leading to systemic inflammation despite combination antiretroviral treatment (cART). This inflammatory milieu promotes T-cell activation and exhaustion. Furthermore, it produces diminished effector functions including loss of cytokine production, cytotoxicity, and proliferation, leading to disease progression. Exhausted T cells show overexpression of immune checkpoint molecules (ICs) on the cell surface, including programmed cell death protein 1 (PD-1), cytotoxic T-lymphocyte-associated antigen-4 (CTLA-4), T-cell immunoglobulin and mucin-domain containing-3 (TIM-3), T-cell immunoglobulin and immunoreceptor tyrosine-based inhibitory motif domain (TIGIT), and lymphocyte activation gene-3 (LAG-3). The ICs also play a crucial role in T-cell exhaustion by reducing the immune response to cancer antigens. Immunotherapy based on immune checkpoint inhibitors (ICIs) has changed the management of a diversity of cancers. Additionally, the interest in exploring this approach in the setting of HIV infection has increased, including AIDS-defining cancers and non-AIDS-defining cancers in PLWH. To date, research on this topic suggests that ICI-based therapies in PLWH could be a safe and effective approach. In this review, we provide an overview of the current literature on the potential role of ICI-based immunotherapy not only in cancer remission in PLWH but also as a therapeutic intervention to restore immune response against HIV, revert HIV latency, and attain a functional cure for HIV infection.

## Introduction

Combination antiretroviral treatment (cART) has significantly improved the immune status of people living with HIV (PLWH) and has dramatically reduced HIV morbidity and mortality, transforming HIV infection into a chronic disease (1). However, cART is not curative and PLWH require lifelong treatment due to the establishment of the HIV reservoir in long-lived CD4 T cells allowing the virus to persist in a quiescent state evading immune detection (2). After many years of research in HIV infection, there is an undeniable need for therapeutic strategies that can enhance antiviral immunity and reduce the viral reservoir that is critical for permanent HIV remission.

Long-term persistence of HIV has been associated with T-cell exhaustion, which consists of T-cell functional impairment with loss of cytokine production, cytotoxicity, proliferation (3), and consequent disease progression (3–5). Exhausted T cells are characterized by an overexpression of immune checkpoint molecules (ICs) on the T-cell surface such as programmed cell death protein 1 (PD-1), cytotoxic T-lymphocyte-associated antigen-4 (CTLA-4), T-cell immunoglobulin and mucin-domain containing-3 (TIM-3), T-cell immunoglobulin and immunoreceptor tyrosine-based inhibitory motif domain (TIGIT), lymphocyte activation gene-3 (LAG-3), CD160, and 2B4 (3, 6–8). Interestingly, Banga et al. have demonstrated that PD-1 expressing memory CD4 T cells from lymph nodes are the main source of infectious viruses in PLWH (9). Similarly, another study reported that in PLWH with uncontrolled viremia, CTLA-4 expressing CD4 T cells contain higher HIV DNA levels compared with their CTLA-4 negative CD4 T-cell counterparts (10). Fromentin et al. have shown that co-expression of immune checkpoint molecules such as PD-1, LAG-3, and TIGIT in CD4 T cells leads to HIV latency during ART regimen (11).

PLWH have an increased risk of developing multiple cancers due to co-infection with certain oncogenic viruses, certain unhealthy lifestyle behaviors, and persistent immune alterations despite cART. These cancers include AIDS-defining cancers, such as non-Hodgkin lymphoma, cervical cancer, or Kaposi sarcoma, as well as non-AIDS-defining cancers such as Hodgkin lymphomas, anal cancer, lung cancer, and hepatocellular carcinoma, among others (12). Furthermore, non-HIV-related hematological malignancies such as acute leukemia, multiple myeloma, and myeloproliferative neoplasms are emerging among PLWH (13).

ICs also play an important role in T-cell exhaustion in cancer patients by decreasing the immune response against cancer antigens (3, 14). Antibodies (Abs) that specifically block ICs, also named immune checkpoint inhibitors (ICIs), including anti-CTLA-4, anti-PD-1, and anti-PD-L1, are transforming cancer therapies by enhancing anti-cancer immunity. This leads to increased survival rates in patients with various types of cancers, even in PLWH. Considering the success of ICIs in cancer therapy and taking into account that T-cell exhaustion in cancer is similar to that observed in HIV infection, the potential role of ICI-based therapies in improving T-cell responses has gained interest. ICIs can be used in the control of HIV infection as an adjuvant to standard cART to improve HIV immune response and revert virus latency.

The relevance of ICI-based therapies, in both cancer and chronic infections including HIV, is reflected in recent reviews addressing various aspects of this field (15–20). In the present review, we give a general overview of the possible impact of ICI-based treatments for cancer remission in PLWH as well as its impact on the HIV-specific immunological response to revert T-cell exhaustion and HIV latency. ICIs could represent a novel form of latency reversion agents (LRAs): on one side reactivating HIV transcription from latently infected cells which will improve HIV recognition by the immune system, while also reinvigorating HIV-specific T-cell responses. Together, this could support the eradication of HIV-infected cells.

## Immune checkpoint molecules and T-cell exhaustion in HIV infection

T-cell exhaustion has been described as a hallmark of several chronic infections. This includes HIV, in which antigens persist with deleterious effects on the proliferative capacity and effector functions of antigen-specific T cells. The immune system has mechanisms to ensure a specific and controlled response to antigens controlling T-cell hyperreactivity through the expression of ICs. This includes the most studied ICs, PD-1 and CTLA-4. Engagement of these molecules with their ligands provides signals counteracting the activation of T cells after T-cell receptor (TCR) stimulation (21, 22). These ICs are upregulated upon ongoing antigen exposure which contributes to a loss of proliferating capacity and reduced production of cytokines by T cells, therefore leading these cells to a state of immune exhaustion. In addition, some studies have shown that T-cell exhaustion also exhibits impaired memory as well as metabolic and transcriptional cell abnormalities (3, 23). Furthermore, it is recognized that exhaustion can occur in immune cell types other than CD8 T cells, such as CD4 T cells (23), NK cells (24), and B cells (25).

PD-1 and its ligand receptors, PD-L1/PD-L2, are the ICs most responsible for T-cell exhaustion in chronic infections. However, other ICs, such as CTLA-4, TIM-3, TIGIT, or LAG-3 can be expressed alone or in combination to cause pronounced T-cell exhaustion. Immune exhaustion is typically characterized by the co-expression of numerous ICs by T cells. Generally, the higher the number of co-expressed ICs by T cells, the more severe the exhaustion (3, 7). Several studies in HIV infection have shown an increased expression of ICs both in CD4 and CD8 T cells. This has been associated with reduced effector functions, less virus-cell clearance, declining CD4 T-cell numbers, and disease progression (4, 5, 8, 26–28).

Unquestionably, cART has significantly prolonged the life expectancy of PLWH. However, it has been noted that suppression of viral replication with cART does not revert the increased expression of ICs such as PD-1, TIM-3, or TIGIT (26, 29), even when cART is initiated in acute infection (30). Notably, in PLWH receiving cART, the expression of PD-1 on T cells is a predictor of viral rebound following cART interruption (31) and has been associated with poor recovery of CD4 T-cell counts (32, 33).

## Immune checkpoint molecules and HIV persistence

The HIV reservoir remains the principal obstacle to achieving eradication of HIV infection despite successful cART. This is mainly due to the presence of resting long-lived memory CD4 T cells harboring persistent replication-competent viruses. The homeostatic proliferation of infected CD4 T cells also contributes to the replenishment of the viral reservoir (34). Interestingly, it has been suggested that T-cell exhaustion is another crucial factor for HIV persistence (35). Khoury et al. have reported a significant correlation between the frequency of PD-1 expression on CD4 T cells and HIV persistence in rectal and blood tissues in PLWH receiving cART (36). Moreover, it has been shown that CD4 T cells expressing PD-1 in blood and lymph nodes from cART-suppressed PLWH are an important source of viral latency (9, 37, 38).

Furthermore, it has been described that the expression of T-cell exhaustion markers, including PD-1 and LAG-3, during acute HIV infection prior to the use of cART, correlates with HIV reservoir size and can predict viral rebound after treatment interruption (31). Consistent with these results, other studies have demonstrated that blood CD4 T cells expressing PD-1 alone (39) or co-expressing LAG-3 and TIGIT (11) showed a high level of integrated HIV DNA. In a recent study, Horn et al. have described that high levels of HIV DNA and PD-1 expression on CD4 T-cell subsets persist in peripheral blood and the terminal ileum of PLWH despite cART (40). Similarly, it has been shown that double-positive PD-1^+^CTLA-4^+^ memory CD4 T cells from the blood of PLWH on cART have a high frequency of HIV DNA^+^ cells, favoring HIV persistence (41). There is still a great deal to be discovered about how ICs induce virus latency. Evans et al. have suggested that the inhibitory effects of ICs, such as PD-1, on T-cell activation lead to a reduction of HIV transcription and RNA translation, therefore promoting HIV latent infection (39). Similar results have been found for CTLA-4, with higher levels of HIV latency in CTLA-4^+^ than in CTLA-4^-^ T cells. Furthermore, downmodulation of CTLA-4 expression on T cells induced by HIV-Nef protein results in transition from latent to productive infection of these cells, supporting the role of CTLA-4 as a modulator of HIV latency (10).

## A glance at therapies based on blocking immune checkpoints molecules in cancer

Exhausted T cells have been found in several malignancies, which create an immunosuppressive environment in tumor tissues, similar to what happens in HIV infection (3). Dysfunctional anti-tumor T-cell responses have been linked to the elevated and sustained expression of numerous ICs (PD-1, CTLA-4, TIM-3, TIGIT, or LAG-3) in a variety of malignancies. These ICs, which regulate T-cell function through various pathways, induce tolerance, exhaustion, and produce inhibitory signals that reduce the immune response against tumor cells (3, 14, 42–44).

The discovery that T-cell exhaustion is a reversible phenomenon has made critical advances in cancer immunotherapy. Therapy based on the blockade of ICs to reinvigorate T-cell responses has revolutionized cancer treatment. ICIs are monoclonal antibodies (mAbs) directed against ICs expressed by immune cells that prevent the inhibition of anti-cancer T cells, primarily CD8 T cells, enhancing the specific responses to tumors. In the past few years, it has been shown how the administration of ICIs can reactivate the exhausted immune system and increase survival in patients with a wide variety of cancers (45).

Several molecules have been approved for the management of advanced malignancies, including non-small cell lung cancer (NSCLC), melanoma, and head and neck cancer, among others (46–49). *Ipilimumab*, an ICI that selectively targets CTLA-4 and has shown promising results in the treatment of melanoma, was the first ICI approved by the FDA for use in cancer immunotherapy (47). Other mAbs for cancer therapy have been approved, including anti-PD-1 (*nivolumab*, *pembrolizumab*, and *cemiplimab*) and anti-PD-L1 (*atezolizumab*, *durvalumab*, and *avelumab*), with anti-tumor activity in several types of cancer, such as melanoma, lung cancer, Hodgkin lymphoma, metastatic anal cancer, cutaneous squamous cell carcinoma, and breast cancer, among others (46, 49–53). Importantly, combined administration of anti-CLTA-4 and anti-PD-1 has demonstrated to be a successful tool for the therapy of several cancers (44, 54–57). Currently, there are next generation ICIs other than CTLA-4 and PD-1/PD-L1. One example is anti-LAG-3 (*relatlimab*) which has been recently approved for metastasic melanoma treatment in combination with *nivolumab* (58). Others such as anti-TIGIT or anti-TIM-3, as well as new combinations of ICIs, are being explored to potentiate anti-tumor immune response in different cancer types including solid tumors, multiple myeloma, endometrial cancer, and gastric cancer, among others (NCT02913313, NCT03119428, NCT02817633, NCT03099109, NCT02608268, NCT01968109).

The inhibiting activity of ICIs on molecules with an immune system regulatory function means that ICI therapies are not without risk of toxicity and may result in a range of immune-related adverse effects (irAEs). The most common irAEs occur in the skin, endocrine system, gut, and lungs. Severe grades of irAEs, such as pneumonitis, neurotoxicity, and cardiovascular and renal toxicity have been well described in cancer patients receiving ICIs. Most of these irAEs can be treated successfully with immunosuppressor drugs, including corticosteroids (59–61). If corticosteroids are ineffective, other treatments such as *infliximab* or *vedolizumab* have been evaluated to treat these ICI side effects (62, 63).

Another aspect to consider when using ICIs for cancer treatment is the safety of their use in patients with specific associated comorbidities, such as concomitant chronic infections (64, 65) or immune-related disorders (64, 66). Overall, there is consensus that cancer patients with different comorbidities may benefit from ICI treatment. Nevertheless, precautions should be taken to reduce the occurrence of major side effects in these patients. Additionally, these precautions vary depending on the specific comorbidity.

## Therapeutic potential of immune checkpoint inhibitors in HIV infection

Given the success of immunotherapy in the treatment for cancer, ICI-based immunotherapy is an intriguing field that is gaining attention as a coadjuvant to improve the immune response to HIV infection. Unfortunately, there is no vaccine or cure for this viral infection, and as stated previously, T-cell exhaustion is a primary characteristic of chronic HIV infection. The unique therapeutic approach that has demonstrated a complete eradication of HIV has been hematopoietic stem cell transplantation. This has occurred in four HIV-infected individuals with hematological malignancies who received a hematopoietic stem cell transplantation from donors homozygous for CCR5Δ32 (67–70). However, hematopoietic stem cell transplantation has a high risk of morbidity and mortality, is too complex, costly, and would therefore be inaccessible as a worldwide therapeutic strategy for the eradication of HIV. The most realistic approach is the functional cure that focuses on developing immunotherapy to boost host immunity and achieve a complete suppression of virus replication without cART. Thus, blocking ICs as a strategy to reinvigorate HIV-specific T-cell responses has raised considerable interest as explained below.

## *Ex vivo* and *in vitro* studies

Different *ex vivo* studies have demonstrated that PD-1/PD-L1 or CTLA-4 blockade in cultured cells from PLWH can reinvigorate the proliferative ability and functionality of HIV-specific CD4 and CD8 T cells (6, 27, 71). Similarly, blockade of PD-1 or TIM-3 molecules restores cytokine production by HIV-specific CD8 T cells (4, 26, 29). Porichis et al. have shown that PD-1 blockade along with IL-10 treatment reinvigorated CD4 T cells and improved NK function, suggesting that inhibition of T-cell exhaustion can reinvigorate not only adaptive but also innate immune responses in HIV (72). Holder et al. reported that treating NK cells from PLWH with anti-TIGIT mAbs increased NK cell activity against HIV-infected CD4 T cells (73). Moreover, a very recent *ex vivo* study using cells from PLWH on ART has demonstrated that the combined blockade of multiple ICs, including PD-1, PD-L1, LAG-3, CTLA-4, TIM-3, and TIGIT, had a synergistic effect increasing the frequency of HIV-specific CD4 and CD8 T-cell responses when compared with the blockade of a single IC (74).

Brunet-Ratnasingh et al. measured the ability of different HIV-specific CD4 T-cell subsets to respond to PD-L1 blockade and found that follicular helper T (Tfh) cells, previously described as an important source of replication-competent HIV (75), showed low responsiveness to PD-L1 blockade while Th1, Th17, and Th22 cells significantly increased their responsiveness (76). This finding highlights the importance of considering the different lineages of HIV-specific CD4 T cells to better evaluate the potential role of ICIs for reverting T-cell dysfunctionality in PLWH.

Another interesting effect of ICIs in PLWH is the reversion of HIV latency in CD4 T cells, as demonstrated in two *ex vivo* studies indicating that blocking the PD-1/PD-L1 pathways with anti-PD-1 antibodies enhanced viral production and resulted in a decrease in HIV latency (39, 77). Also, an *in vitro* study has demonstrated that when compared to other commonly used latency-reverting drugs such as *vorinostat* and *bryostatin*, the level of latency reversion following simultaneous blockade of different ICs such as PD-1, CTLA-4, TIGIT, and TIM-3 was much higher (78).

## Preclinical studies

Studies in animal models of simian immunodeficiency virus (SIV) infection have demonstrated that PD-1 or PD-L1 blockade resulted in enhanced virus-specific CD8 T-cell effector functions (79, 80) and reduced viral load (79). In ART-treated SIV-infection, the PD-1 blockade resulted in a reduction of replication-competent virus reservoirs as well as increased expansion of CXCR5-expressing and granzyme-B-producing CD8 T cells. It also offered better control of SIV-viremia following ART interruption (81). Very recently, it has been reported that *in vivo* PD-1 blockade after ART interruption in SIV-infected macaques was able to: a) enhance SIV-specific CD8 T-cell functions; b) induce an expansion of memory CD4 and CD8 T cells and cytolytic NK cells; and c) control viremia after interruption of ART (82). Combinatorial approaches including blockade of PD-1 and LAG-3 (7) or PD-1 and TIM-3 (83) in mice have shown that these dual blockades were able to reinvigorate CD8 T-cell responses. Importantly, in ART-treated SIV-infection the dual CTLA-4/PD-1 blockade was able to reduce the SIV reservoir size (84). This result is in accordance with another study in which blockade of CTLA-4 led to increased viral replication at mucosal sites of SIV-infected macaques (85).

**Figure 1:**
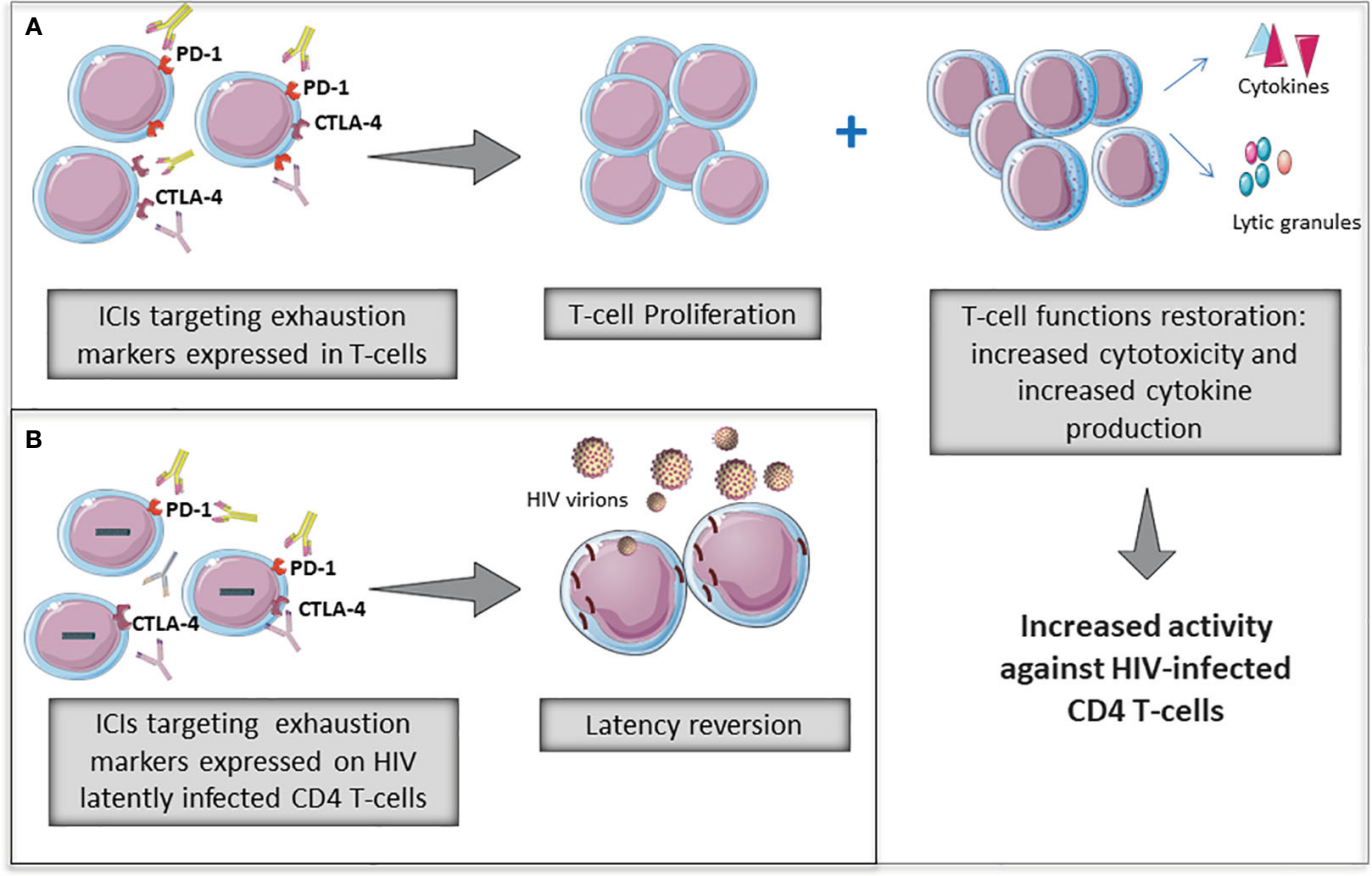
Potential role of immune checkpoint inhibitors (ICIs) to revert T-cell exhaustion **(A)** and to revert HIV latency **(B)** in PLWH. Some ICIs widely used in cancer therapies are being evaluated for restorating T-cell functions as well as reverting HIV-latency in PLWH.

Overall, the above-mentioned studies provide evidence that the blockade of some ICs, such as anti-PD-1 and/or CTLA-4 could have the potential to reverse T-cell exhaustion by improving HIV-specific immune functionality and to revert HIV-latency in PLWH (**Figure 1**).

**Table 1** summarizes the *ex vivo* and *in vivo* findings reported to date focused on the role of immune checkpoint blockade in the setting of HIV/SIV infections.

**Table 1:** Summary of *ex vivo* and *in vivo* studies reporting immune checkpoint blockade in the setting of HIV/SIV infections.

IC molecule	Effect of *ex vivo* blockade	Effect of *in vivo* blockade
**PD-1**	- Reinvigorated CD4 T-cells functionality and enhanced NK functions (72) - Reduced HIV latent infection (39, 77)	- Reduced viral reservoir and increased expansion of CD8 T cells expressing CXCR5 and granzyme-B in SIV infection (81) - Enhanced SIV-specific CD8 T-cell responses and B-cell responses as well as reduced viral load and increased survival (79) - Enhanced SIV-specific CD8 T-cell responses as well as proliferation of memory CD4 and CD8 T cells and cytolytic NK cells (82) - Reverted HIV-latency (39, 86, 87) as well as mildly increased HIV-specific CD8 T-cell responses (86) - Increased virus-specific CD8 T-cell response after PD-1 blockade alone (88) or in combination with anti- CTLA-4 (89)
**PD-L1**	- Increased the proliferation of HIV-specific CD8 T cells (71) - Increased responsiveness of HIV-specific Th1, Th17, and Th22 (76)	- Increased SIV-specific CD8 and CD4 T-cell responses (80) - Increased of HIV specific CD8 T-cell responses (90)
**CTLA-4**	- Increased activity of NK against HIV-infected CD4 T cells (73) -	- Reverted HIV-latency (91) - Reverted SIV-latency after dual blockade of CTLA-4 and PD1 (84) - Reverted HIV-latency after dual blockade of PD-1 and CTLA-4 (89, 92) and also increased HIV-specific CD8 T-cell response (89)
**TIGIT**	- Restored HIV-specific CD8 T-cell response after dual blockade of PD-1 and TIGIT (8) - Reverted HIV-latency with multiple blockade of ICs: PD-1, CTLA-4, TIGIT, and TIM-3 (78)	- Increased IL-2 production by T cells in SIV infection after dual blockade of TIGIT and PD-1 (8)
**TIM-3**	- Increased proliferation of HIV-specific T-cell responses (29) - Increased HIV-specific CD8 T-cell responses with dual blockade of TIM-3 and PD-1 (83)	- Reinvigorated CD8 T-cell responses in mice after dual blockade of TIM-3 and PD-1 (83)
**LAG-3**	- Increased frequency of HIV-specific CD4 and CD8 T cells after blockade of LAG-3 and other ICs (74)	- Reinvigorated CD8 T-cell responses in mice after dual blockade of LAG-3 and PD-1 (7)

PD-1, programmed cell death protein 1; PD-L1, programmed cell death protein ligand 1; CTLA-4, cytotoxic T-lymphocyte-associated antigen-4; TIGIT, T-cell immunoglobulin and immunoreceptor tyrosine-based inhibitory motif domain; TIM-3, T-cell immunoglobulin and mucin-domain containing-3; LAG3, lymphocyte activation gene-3.

## ICIs in PLWH with malignancies

The knowledge regarding ICI-based therapies could also be applied to PLWH with cancer, with the benefit that several ICIs have previously been established for cancer therapy with successful outcomes. However, most clinical trials studying ICIs in cancer patients have excluded patients with chronic infections such as HIV based on the concern that this population lacks sufficient T-cell immunity to benefit from therapy, as well as the risk of potential severe side effects associated with reestablishing HIV immunity with this therapy. Fortunately, accumulated experience to date from case series and clinical trials has shown that ICIs are safe in PLWH and that the incidence of adverse effects (irAEs) is not higher than in the general non-HIV-infected population, as discussed below. Only some of these studies have reported some incidence of irAEs in PLWH treated with ICIs, including hypothyroidism, pneumonitis, rash, uveitis, and allergic lung disease (93–95). Importantly, thyroiditis and autoimmune hepatitis were observed in a trial with anti-PD1, leading to the early termination of the trial (96).

Although the information about the benefits and safety of ICIs in the treatment of malignancies in PLWH is still scarce, there are some case series, retrospective cases, and cohort reports on PLWH diagnosed with malignancies and treated with ICIs. Two recent systematic reviews have summarized the case reports evaluating the safety and efficacy of ICIs in PLWH, as well as the impact in HIV progression markers in some of the studies (97, 98). Overall, the use of ICIs in PLWH was safe, and rates of adverse events were between 9% to 12%. These percentages were similar to those observed in cancer therapies in the HIV-negative population (97, 98). Regarding efficacy of ICIs against cancer in PLWH, it was highly variable depending on the tumor type, with response rates varying from 27% to 63% (97). In addition, the effect of ICIs on HIV disease markers was very modest, with 24% to 56% of patients showing increases in CD4 cell count (97, 98). In addition, data on HIV reservoirs and HIV-specific responses was available only in 7% (13/176) of PLWH of which 23% (3/13) showed improvement of HIV-specific CD8 T-cell response, and 15% (2/13) showed a decrease of HIV reservoir (98).

Interestingly, the most recent data from a retrospective study by El Zarif et al. have provided insight on the safety of ICIs among 390 PLWH with cancer that were treated with anti-PD1 or anti-PD-L1. This study demonstrated minimal ICI-related adverse events: 20% of patients had irAEs of any grade, and only 8% of patients had high grade irAEs. These data support that ICIs are safe to use in PLWH and can therefore benefit from this treatment (99).

Among the different ICIs, the most investigated in the context of PLWH with malignancies have been anti-PD-1 and anti-PD-L1. Several studies, ranging from case reports to multicenter cohorts, have demonstrated the safety of anti-PD-1 and anti-PD-L1 in PLWH suffering from different cancers. Idossa et al. and Alloghbi et al. reported three cases of PLWH on cART treated with anti-PD-1 for metastatic prostate cancer (treatment with *pembrolizumab* for two cases) and for advanced cutaneous squamous cell carcinoma (treatment with *cemiplimab* for one case), respectively. All patients responded to therapy and showed no major toxicities (100, 101).

Additionally, in two different case series studies of PLWH on cART and treated for a variety of malignancies, an excellent tolerability of anti-PD-1 and/or anti-PD-L1 therapy was observed, and all patients showed stable CD4 T-cell counts and no reactivation of HIV load during ICI therapy (93, 94). Similar results, with no evidence of ICI-related serious irAEs or negative effects on CD4 counts or viral load, were observed in two multicenter studies enrolling 20 and 30 PLWH on cART, respectively, one using anti-PD-1 (95) and the other anti-PD-L1 (102). Furthermore, a study by Galanina et al. showed an increase of CD4 T-cell counts in nine PLWH on cART who received anti-PD-1 for treating Kaposi sarcoma (103).

In addition, some of the case reports published to date have provided evidence that PLWH on cART who have received ICIs such as anti-CTLA-4 (91) or anti-PD-1 (39) for treatment of melanoma can display an increase in HIV transcription (measured by HIV-RNA in CD4 T cells), suggesting an *in vivo* effect of these therapies in the reversion of virus latency. Moreover, two different studies in PLWH on cART treated with anti-PD-1 for lung cancer showed not only a decrease in HIV reservoir size but also a decrease of CD4 and CD8 T-cell exhaustion with an increase in HIV-specific CD8 T-cell response (86, 87). Another case report in PLWH on cART with melanoma showed enhancement of HIV-specific CD8 T-cell response after treatment with the anti-PD-1 *pembrolizumab* (88). Interestingly, Lau et al. evaluated the impact on HIV-specific response and changes in HIV reservoir in three PLWH on cART after receiving repeated cycles of different ICIs (anti-PD-1, anti-PD-L1, anti-CTLA-4, or a combination of some of them) depending on their specific malignancy. These authors found that all participants showed some degree of HIV-latency reversion (evidenced by increased cell-associated unspliced HIV RNA). However, a decrease in the HIV reservoir was observed in only one patient, and an increase in virus-specific CD8 T-cell responses in another (89). On the other hand, Baron et al. reported a limited effect on the HIV reservoir using monotherapy with anti-PD-1 in PLWH cART with cancer, with a concomitant increase in expression of other ICs on T cells, suggesting the existence of compensatory mechanisms limiting the efficacy of ICI monotherapy (104). This suggests that combination therapy with several ICIs may be a better strategy to pursue a cure for HIV.

It is important to note that, currently, there are only a few clinical trials being carried out in PLWH with advanced-stage cancer (**Table 2**). All of these studies are in their early phases: the first is the AIDS Malignancy Consortium (AMC) 095 study, a phase I trial (NCT02408861) evaluating the safety and optimal dose of therapy with *nivolumab* (targeting PD-1) alone or in combination with *ipilimumab* (targeting CTLA-4) in PLWH on cART with advanced solid tumors. Interestingly, a substudy of this clinical trial showed that combination therapy with anti-PD-1 and anti-CTLA-4 in seven of the participants was able to revert HIV latency and in two patients was able to decrease cells containing replication-competent virus (as estimated by the quantitative viral outgrowth assay (QVOA)). These findings suggest a synergistic effect of dual therapy of ICIs on HIV latency reversion and support the hypothesis that combination therapy with several ICIs could be useful to significantly impact HIV reservoirs (92).

**Table 2:** Summary of ongoing clinical trials on immune checkpoint therapies in PLWH with malignancies.

Targeting IC molecule	NCT number	Outcome measures related with HIV infection	Intervention/ Treatment	Study type/Phase
PD-1 CTLA-4	NCT02408861	**In PLWH with advanced solid tumors**: • Maximum dose of nivolumab • Immuno-virological evolution of HIV infection (HIV-RNA load, T-cell counts, and HIV reactive cells)	*Nivolumab* and *ipilimumab*	Interventional/Phase I
PD-1	NCT02595866	**In PLWH with refractory, relapsed, or disseminated malignant neoplasms**: • Frequency of AEs • Frequency of irAEs • Frequency of ART-related ECIs • Proportion of patients who achieve complete or partial response • Progression-free and overall survival	*Pembrolizumab*	Interventional/Phase I
PD-1	NCT04929028	**In PLWH with high risk and low risk HIV-associated anal cancer**: • Frequency of AEs • Change in CD4 T-cell counts and HIV-load • Changes in cART adherence	*Nivolumab* combined with chemotherapy and radiotherapy	Interventional/Phase II
PD-1 CTLA-4	NCT03354936	**In PLWH with cancer (any)**: • Frequency of clinical and biological AEs • Overall response rate, progression free survival rate • Immuno-virological evolution of HIV-infection (CD4 and CD8 T-cell counts, HIV-RNA load, residual plasma HIV-RNA, HIV-specific T-cell responses, and inflammation/activation/exhaustion markers)	*Nivolumab*, *pembrolizumab*, and *ipilimumab*	Observational
PD-1	NCT03304093	**In PLWH with NSCLC:** • Disease control rate • Progression free survival/overall survival • Frequency of AEs • Duration of response • Immuno-virological evolution of HIV- infection (HIV-RNA load, HIV-DNA, residual HIV replication, and T-cell activation/exhaustion markers)	*Nivolumab*	Interventional/Phase II
PD-L1	NCT04499053	**In PLWH with NSCLC:** • Frequency of AEs • Radiological response • Blood tumor mutational burden • Immuno-virological evolution of HIV- infection (HIV-RNA load, cytokine profile, and immune biomarkers)	*Durvalumab* combined with chemotherapy	Interventional/Phase II
PD-1	NCT04514484	**In PLWH with advanced solid tumors:** • Frequency of dose limiting toxicities • Immuno-virological evolution of HIV- infection (HIV-RNA load, CD4 and CD8 T-cell counts, activation and exhaustion markers) • Changes in infiltrating immune cell markers and angiogenesis markers	*Nivolumab* combined with *cabozantinib*	Interventional/Phase I

PLWH, people living with HIV; PD-1, programmed cell death protein 1; PD-L1, programmed cell death protein ligand 1; CTLA-4, cytotoxic T-lymphocyte-associated antigen-4; AEs, adverse events; irAEs, immunological-related adverse events; ECI, events of clinical interest; NSCLC: non-small cells lung carcinoma; CSF, cerebrospinal fluid.

The second study, a phase I clinical trial (NCT02595866) from the Cancer Immunotherapy Trials Network [(CITN)-12 trial] is still recruiting PLWH on cART with relapsed malignant neoplasms to evaluate the side effects of *pembrolizumab*. In a sub-study with 32 participants of this trial, Uldrick et al. reported that the PD-1 blockade slightly reverted HIV latency after the first cycle of anti-PD-1 treatment (105). There are two other ongoing trials; one (NCT03354936) is aimed to evaluate the safety of *nivolumab* or *pembrolizumab* (anti-PD-1) combined with *ipilimumab* (anti-CTLA-4) in PLWH with cancer; the other trial (NCT03304093) evaluates both the safety and efficacy of *nivolumab* in PLWH on cART with non-small cell lung cancer (NSCLC).

Finally, there are other ongoing clinical trials that aim to evaluate the side effects of combination therapy of ICIs with other types of anti-cancer drugs in PLWH on cART. Among these trials are: **a)** the phase I trial NCT04514484 assessing the side effects of the combination of *nivolumab* with *cabozantinib* (a kinase inhibitor) in PLWH with different types of malignancies; **b)** the phase II trial NCT04929028 that studies the side effects of *nivolumab* in combination with different chemotherapy drugs (e.g., *capecitabine* and *fluorouracil*) in participants with ADIS-related anal carcinoma; **c)** the phase II trial NCT04499053 that evaluates adverse events in PLWH with NSCLC after treatment with *durvalumab* (anti-PD-L1) and platinium-based chemotherapy; and d) the phase I trial NCT04902443 that investigates the safety and tolerability of combined therapy of *nivolumab* plus *pomalidomide* in PLWH with virus-associated malignancies.

Taken together, the data provided by the above mentioned studies, while limited and variable, clearly advocates for the use of ICI therapy in the PLWH population suffering from cancer. Moreover, ICI therapy in PLWH could have an additional benefit on HIV disease parameters, restoring virus-specific T-cell responses and reactivating virus production from the reservoir, mimicking the “shock and kill” concept proposed as one of the main strategies to purge the viral reservoir and cure HIV infection.

## ICIs in PLWH without malignancies

Regarding the novelty and interesting field of the use of ICIs for HIV treatment in PLWH without cancer, the safety and/or efficacy of ICIs has been evaluated in some clinical trials. Two of the trials have already published results: **a)** the phase I trial (NCT02028403) has demonstrated that PD-L1 blockade with the BMS-936559 molecule was well tolerated and induced an increase of HIV-specific polyfunctional CD8 T-cell responses in PLWH on cART (90); **b)** the phase I trial (NCT03407105) using the anti-CTLA-4 *ipilimumab* in PLWH with uncontrolled viremia did not observe significant changes in CD4 T-cell counts and only a moderate decrease of HIV-RNA load after ICI treatment (106); **c)** the phase I/IIa double-blind placebo-controlled trial (NCT03787095) in PLWH on cART evaluated the efficacy of *cemiplimab* (anti-PD-1) and the functional profile of HIV Gag-specific CD8 T-cell responses. However, this study was stopped because two out of four participants showed irAEs at the lowest dose of *cemiplimab* (96).

Moreover, there are four ongoing clinical trials with anti-PD-1 in PLWH on cART without cancer (**Table 3**). The first trial (NCT03239899) evaluates the safety and tolerability of *pembrolizumab* in PLWH as well as its effect on viral load, CD4 T-cell counts, T-cell phenotype, HIV-specific T-cell responses, and cytokine and antibody profile on cerebrospinal fluid (CSF). The second trial (NCT03367754) is recruiting PLWH on cART with poor CD4 recovery and will evaluate, as the primary outcome, the safety of a single dose of *pembrolizumab*, and as the secondary outcome the changes in PD-1 expression on T cells. The third, a phase I/II trial (NCT05187429), is studying the safety of a single dose of *nivolumab* as the primary outcome, as well as the effect on PD-1 expression on T cells, HIV-specific T-cell responses, and viral rebound after analytical treatment interruption (ATI) as the secondary outcome in PLWH. Finally, the fourth trial (NCT04223804) is a double-blind placebo-controlled phase I trial that will test the pharmacokinetics and pharmacodynamics of different doses of *budigalimab* (ABBV-181, anti-PD-1) in PLWH on ART willing to undergo an ATI.

**Table 3:** Summary of ongoing clinical trials on immune checkpoint therapies in PLWH without malignancies.

Targeting IC molecule	NCT number	Outcome Measures	Intervention/ Treatment	Study type/Phase
PD-1	NCT03239899	• Safety and tolerability of a single dose of pembrolizumab • Changes in HIV-specific antibody responses in CSF • Changes in cytokine profile of CSF	*Pembrolizumab*	Interventional/Phase I
PD-1	NCT03367754	• Frequency of AEs • Changes in magnitude of T-cell expression of PD-1	*Pembrolizumab*	Interventional/Phase I
PD-1	NCT04223804	• Frequency of AEs grade 3 or higher and irAEs • Safety, pharmacokinetics, and pharmacodynamics of multiple doses of drug	*Budigalimab*	Interventional/Phase I
PD-1	NCT05187429	• Frequency and severity of AEs • PD-1 receptor occupancy in T cells from peripheral blood and inguinal lymph node • HIV-specific CD4 and CD8 T-cell responses • Changes in HIV-RNA load during ATI	*Nivolumab*	Interventional/Phase I and Phase II

PLWH, people living with HIV; PD-1, programmed cell death protein 1; PD-L1, programmed cell death protein ligand 1; CTLA-4, cytotoxic T-lymphocyte-associated antigen-4; AEs, adverse events; irAEs, immunological-related adverse events; ATI, analytical treatment interruption; CSF, cerebrospinal fluid.

## Concluding remarks

There is compelling evidence regarding the role of ICs in the attrition of HIV-specific T-cell responses, poor CD4 T-cell recovery, and the viral persistence in PLWH despite cART. Over the past years, there have been many interesting findings related to understanding T-cell exhaustion and how to reinvigorate the HIV-specific immune response by employing mAbs blocking ICs. Without a doubt, persistent HIV latency remains the main obstacle for achieving HIV remission, and different approaches targeting this latency are under intensive investigation. Based on new data, ICI-based therapies used for cancer could be considered not only helpful to target cancer in PLWH but also beneficial to combat immune exhaustion/dysfunction and HIV-latency in PLWH. This approach could be especially attractive as adjuvant therapy in combination with cART with the goal to attain better long-term health outcomes in PLWH. The results provided by current studies are still modest, and further investigation is warranted to accurately measure the effectiveness of this approach in HIV functional cure or remission. Nevertheless, results from ongoing clinical trials will significantly contribute to clarifying the ability of ICI-based therapies to restore HIV-specific immune response and revert HIV latency.

## Search strategy and selection criteria

Relevant scientific literature was surveyed to review evidence and prepare the manuscript. We searched PubMed for English language papers published until September 2023. Search terms included: “HIV disease progression”; “HIV pathogenesis”; “immune exhaustion and HIV”; “immune exhaustion and cancer”; “HIV cure”; “HIV and inflammation”; “AIDS-defining cancers”; “non-AIDS-defining cancers”; “ immune checkpoint molecules and HIV”; “ immune checkpoint molecules and cancer”; “IC and HIV”; “IC and cancer”; “immune checkpoint inhibitors and HIV”; “immune checkpoint inhibitors and cancer”; “immune checkpoint blockers and HIV”; “immune checkpoint blockers and cancer”; “ICIs and HIV”; “ICIs and Cancer”; and “ICIs and HIV and cancer”. Two authors (JMB and CR) screened abstracts for relevance and reviewed full-text articles deemed relevant to the topics addressed in the manuscript. In addition, we searched the database ClinicalTrials.gov for clinical registered trials related to immune checkpoint inhibitors. Search terms included: “HIV and ICIs”; “HIV and anti-PD1”; “HIV and anti-PD-L1”; “HIV and anti-CTLA-4”; “HIV and anti-TIGIT”; “HIV and anti-Tim-3”; and “HIV and anti-LAG-3”.

## Author contributions

JB: Writing – original draft, Methodology, Writing – review & editing. CR: Writing – original draft, Methodology, Writing – review & editing. JG-F: Investigation, Visualization, Writing – review & editing. NR: Conceptualization, Investigation, Funding acquisition, Project administration, Supervision, Data curation, Writing – original draft, Validation, Visualization, Writing – review & editing.
